# Genome-Wide Analysis of GRETCHEN HAGEN3 Genes and Characterization of IAA-Amido Synthetase Gene *CsGH3.1* in Rhizome Proliferation in *Cymbidium sinense* ‘Qijianbaimo’

**DOI:** 10.3390/plants14091287

**Published:** 2025-04-24

**Authors:** Sisi Jia, Cuiling Liu, Chushu Liao, Ruizhen Zeng, Li Xie, Qian Wei, Zhisheng Zhang

**Affiliations:** Guangdong Province Key Laboratory of Plant Molecular Breeding, College of Forestry and Landscape Architecture, South China Agricultural University, Guangzhou 510642, China; 15102080177@163.com (S.J.); 13414854192@163.com (C.L.); zengrz@scau.edu.cn (R.Z.); xieli@scau.edu.cn (L.X.)

**Keywords:** *CsGH3*, *Cymbidium sinense*, rhizome proliferation

## Abstract

This study systematically identified and functionally analyzed the *GRETCHEN HAGEN* 3 (*GH3*) gene family in *Cymbidium sinense* ‘Qijianbaimo’ (CSQ). Seven *GH3* genes (*CsGH3.1*–*CsGH3.7*) were identified using genomic and transcriptomic data. Phylogenetic analysis revealed that CsGH3s are divided into two subfamilies, with CsGH3.1–CsGH3.4 belonging to subfamily II and CsGH3.5–CsGH3.7 to subfamily I. Gene structure analysis showed that *CsGH3s* contain one to four exons, and their promoter regions feature auxin and jasmonic acid response elements. *CsGH3.1* and *CsGH3.2* were significantly upregulated in response to naphthaleneacetic acid (NAA) during rhizome proliferation, indicating their pivotal role in auxin homeostasis regulation. The overexpression of *CsGH3.1* in *Arabidopsis* led to restricted growth and a significant reduction in callus proliferation rates, further confirming its role in regulating cell proliferation and auxin homeostasis. This study provides new insights into the function of the *CsGH3.1* gene in the rhizome proliferation of *Cymbidium sinense* and lays a foundation for future research on the molecular mechanism of micropropagation in *Cymbidium*.

## 1. Introduction

Plants regulate their growth and development in response to environmental signals, with hormones playing a key role, particularly in modulating auxin/indoleacetic acid (IAA) activity and content [1]. The IAA content in specific plant tissues is determined by various metabolic processes, including synthesis, transport, inactivation, reactivation, and degradation through oxidative pathways [2]. In addition to its free form, IAA exists in various modified forms, such as glucose conjugates and amide conjugates with amino acids and peptides [3]. The *GRETCHEN HAGEN* 3 (*GH3*) gene family, a acyl adenylate synthetase family member, is classified into three subfamilies based on similarities in protein sequences and substrate specificity [4]. Subfamily II *GH3* genes are involved in auxin conjugation, catalyzing the binding of IAA to amino acids to form conjugated IAA. In contrast, subfamily I *GH3* genes exhibit adenylation activity for jasmonic acid and catalyze the binding of jasmonic acid to isoleucine [5,6]. As one of the early auxin-responsive gene families, subfamily II *GH3* genes play an important role in regulating auxin homeostasis [7]. When IAA concentrations increase, free IAA is catalyzed by GH3 amido synthetases to bind with amino acids, forming storage forms like IAA-Asp and IAA-Glu. These conjugates can either be converted back to free IAA via ILR1/ILL amido hydrolases or degraded through the *GH3-ILR1-DAO* pathway [6]. This interconversion between free and conjugated IAA helps maintain a dynamic balance in IAA levels.

The *GH3* gene family typically includes multiple members across various plant species. In *Arabidopsis*, 19 *GH3* genes have been identified on chromosomes 1, 2, 4, and 5 [6]. Similarly, 13 *GH3* genes have been identified in rice, located on chromosomes 1, 5, 6, and 7 [8,9]. The *GH3* gene family is also present in other plants such as maize, rapeseed, tomato, coffee, citrus, and potato [10,11,12,13,14]. In *Arabidopsis*, the expression of *AtGH3.2/YDK1*, *AtGH3.5/AtGH3a/WES1*, *AtGH3.6/DFL1*, and *AtGH3.10/DFL2* is regulated by auxin. The overexpression of these genes results in dwarfism, reduced free auxin levels, and increased conjugated IAA-Asp levels [15,16,17,18]. The *AtGH3.17/VAS2* knockout mutant (*vas2*) exhibits elongated hypocotyls and increased free IAA levels [2]. In rice, the overexpression of *OsGH3.1* downregulates genes related to morphogenesis, cell differentiation, and metabolism [19], *OsGH3.2* catalyzes auxin conjugation in response to drought stress [20,21], *OsGH3.8* catalyzes the formation of IAA-Asp from IAA, Asp, and ATP, and the gain-of-function mutant *tld1*-D of *OsGH3.13/TLD1* shows reduced free auxin levels, dwarfism, and decreased fertility [22,23,24,25].

Orchid tissue culture and rapid propagation are core technologies for orchid industrialization, primarily involving the induction, proliferation, and differentiation of intermediate propagules [26]. In terrestrial orchids, the rhizome is the intermediate propagule, but it proliferates slowly and is challenging to differentiate [26]. *Cymbidium sinense* ‘Qijianbaimo’ (CSQ), an important native flower in Guangdong Province, holds a high economic and ornamental value. Enhancing its regeneration capacity is crucial for large-scale propagation. Plant hormones play a vital role in embryogenesis, organogenesis, and plant regeneration [27]. The recently completed genome sequencing of Cymbidium sinense provides a valuable resource for investigating the molecular mechanisms underlying hormone regulation [28]. In tissue culture, exogenous plant hormones facilitate in vitro regeneration by activating specific developmental pathways [29]. Previous studies have shown that adding auxins such as naphthaleneacetic acid (NAA), indolebutyric acid (IBA), and IAA to the medium significantly increases rhizome proliferation rates. A preliminary transcriptomic analysis of *CSQ* during proliferation indicated that *GH3* genes are highly expressed after exogenous NAA application, leading to a deceleration in the rate of endogenous auxin accumulation and limiting rhizome proliferation [26]. The *GH3* genes, known for their role in conjugating endogenous auxin, likely play a significant inhibitory function during the proliferation of CSQ rhizomes, contributing to the observed slow proliferation phenotype.

To further investigate the role of *GH3* genes in orchid tissue culture and rapid propagation, we conducted a comprehensive analysis of the *CsGH3* gene family using the transcriptomic and genomic databases of CSQ, which identified seven *CsGH3* genes. This study examines their phylogenetic relationships, conserved motifs, gene and protein structures, promoter cis-elements, subcellular localization, and expression profiles in response to exogenous auxin during rhizome proliferation. Furthermore, the *CsGH3.1* gene was cloned, and transgenic *Arabidopsis* materials were generated through genetic transformation, confirming the auxin conjugation function of *CsGH3.1* in explant proliferation. These findings provide a theoretical foundation for further research on the role of *GH3* genes in orchid tissue culture, rapid propagation, and auxin-regulated propagation characteristics.

## 2. Results

### 2.1. The Genome of CSQ Contains Seven GH3 Genes

To obtain the *GH3* protein sequences of CSQ, sequence alignment and protein domain analysis were performed using *GH3* protein sequences from *Arabidopsis* and rice. Seven *GH3* protein sequences were identified from the transcriptomic and genomic data of CSQ, located on chromosomes 2, 8, 9, 13, 14, and 15, and named *CsGH3.1*–*CsGH3.7* (Table 1). The chemical properties of the predicted *CsGH3* proteins, including molecular weight (MW), isoelectric point (IP), and chemical formula, were calculated using ProtParam (https://web.expasy.org/protparam// (accessed on 21 October 2023)). The molecular weights of all the *CsGH3* proteins ranged from 65,500 to 69,000 Da, and their isoelectric points ranged from 5.0 to 7.0.

### 2.2. Phylogenetic Relationship of CsGH3s in CSQ

**Figure 1:**
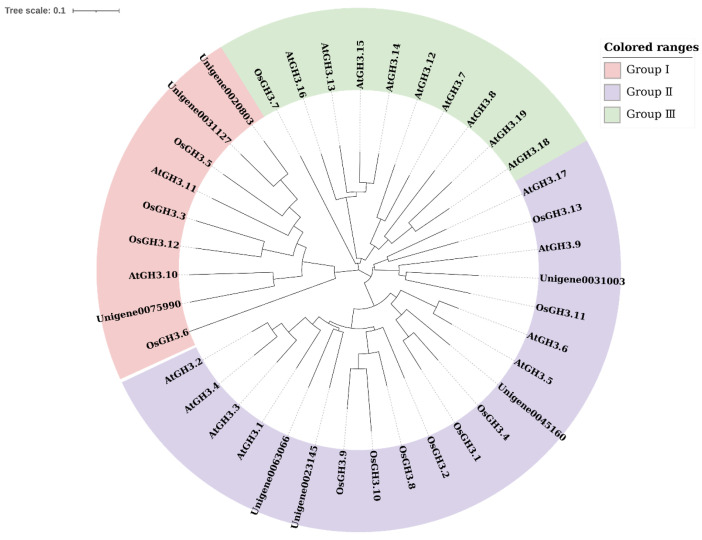
Phylogenetic analysis of *GH3* gene family in CSQ genome.

To study the evolution of the *CsGH3* gene family, a Neighbor-Joining tree was constructed using the protein sequences of the 7 *CsGH3s* from CSQ, 19 *AtGH3s* from *Arabidopsis*, and 13 *OsGH3s* from rice (Figure 1). The *GH3* gene family was divided into three clades in *Arabidopsis*, rice, and other plants [29]. The *CsGH3s* were found in subfamily I and subfamily II, with *CsGH3.1*-*CsGH3.4* belonging to subfamily II (related to auxin conjugation) and *CsGH3.5*-*CsGH3.7* belonging to subfamily I (related to jasmonic acid conjugation). The isoelectric points of the *CsGH3* proteins also varied significantly between subfamilies, with subfamily I proteins showing isoelectric points between 5.5 and 5.8, and subfamily II proteins ranging from 6.2 to 6.3.

Clustal W was used for multiple sequence alignment. MEGA 7.0 was adopted for phylogenetic reconstruction using the Neighbor-Joining (NJ) clustering method. Circles of different colors represent different subfamilies.

### 2.3. Analysis of CsGH3 Gene Structures and Cis Elements in the CsGH3 Gene Promoters

**Figure 2:**
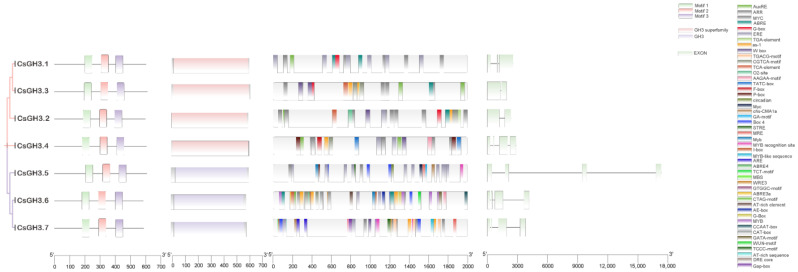
Distribution of conserved motifs, exons, in group II GH3s in *Cymbidium*.

To further study the diversity of *CsGH3* protein structures and predict their functions, conserved motifs and domains were analyzed using the MEME 5.5.7 online software and the NCBI CDD website. The results revealed that all *CsGH3* proteins contained three motifs. Specifically, *CsGH3.1*–*CsGH3.4* from subfamily II exhibited complete *GH3* superfamily conserved domains, while *CsGH3.5*–*CsGH3.7* from subfamily I contained only the *GH3* conserved domain (Figure 2). To predict the potential roles of *CsGH3* family genes at different developmental stages, cis-elements in their promoter regions were identified using the PlantCARE database. The promoters of the *CsGH3* genes contained many plant hormone response elements, including those related to auxin, jasmonic acid, gibberellin, cytokinin, salicylic acid, and abscisic acid (Figure 2). Gene structure analysis showed that the *CsGH3* genes contained between one and four exons. *CsGH3.1* contained three exons, *CsGH3.2* and *CsGH3.3* contained two exons, and *CsGH3.4*–*CsGH3.7* had four exons (Figure 2).

### 2.4. Function of CsGH3 Genes During Rhizome Proliferation in CSQ

**Figure 3:**
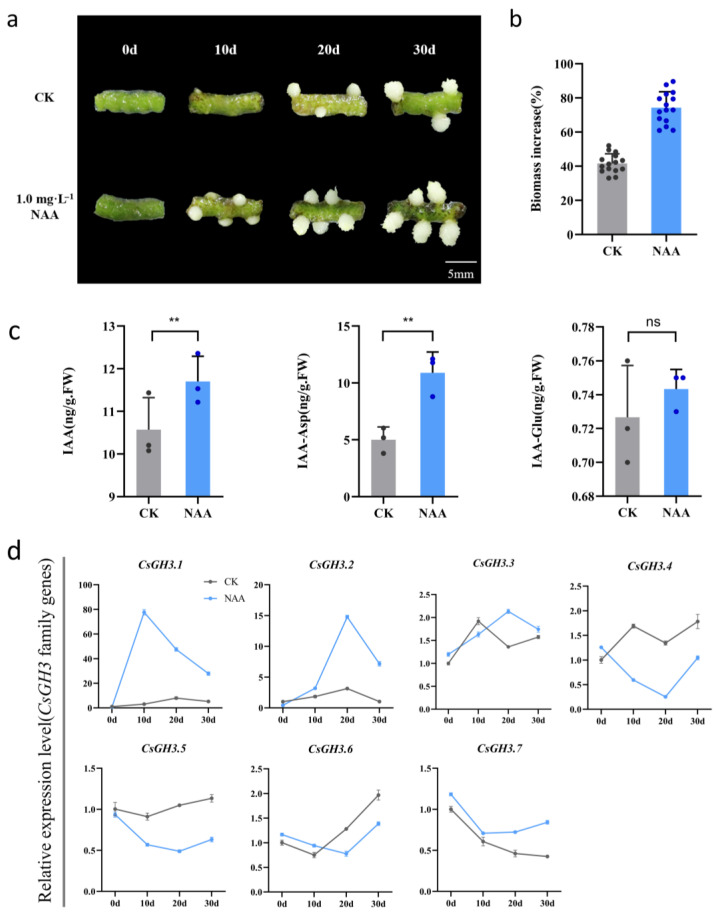
Function of *GH3* gene on rhizome proliferation in CSQ. (**a**) Photograph of CSQ rhizomes cultured on MS medium with 1.0 mg·L^−1^ or without NAA for 0, 10, 20, and 30 days under darkness. Bar = 5 mm. (**b**) Rhizome biomass increment. (**c**) Contents of IAA and IAA-Asp/IAA-Glu in CSQ rhizome. (**d**) Relative expression level of *GH3* genes in rhizome. Values are means ± SD. ** *p* < 0.01; ns, no significance (Student’s *t*-test).

To study the function of the *CsGH3* genes during rhizome proliferation in *CSQ*, the proliferation rate, auxin level, and gene expression level of *CsGH3* were analyzed in rhizomes cultured on an MS medium with and without 1.0 mg·L^−1^ NAA (Figure 3). The results showed a significant increase in the proliferation rate of rhizomes after the exogenous application of 1.0 mg·L^−1^ NAA compared to the hormone-free control (Figure 3a,b). Hormone level measurements revealed that exogenous auxin did not significantly increase the free IAA levels during rhizome proliferation, but the conjugated IAA-Asp levels significantly increased (Figure 3c). These findings suggest that CSQ may maintain auxin homeostasis by converting free IAA into conjugated IAA-Asp during rhizome proliferation. Further analysis of *CsGH3* gene expression in rhizomes under hormone-free and 1.0 mg·L^−1^ NAA conditions showed that *CsGH3.1* and *CsGH3.2* were significantly upregulated at 10, 20, and 30 days in response to NAA, with *CsGH3.1* showing a faster and more pronounced increase in expression (Figure 3d). These results indicate that *CsGH3.1* is crucial in auxin homeostasis during rhizome proliferation.

### 2.5. Overexpression of CsGH3.1 Leads to Decrease in Callus Proliferation Rate in Arabidopsis

**Figure 4:**
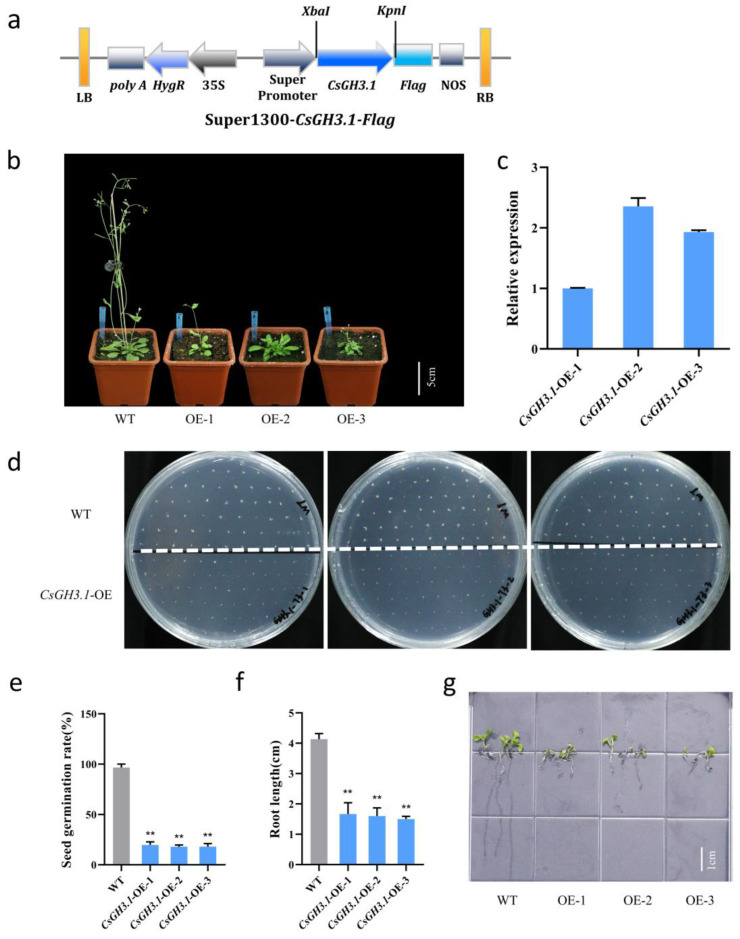
Construction and phenotypic identification of *CsGH3.1* overexpression Arabidopsis lines. (**a**) Schematic of overexpression vector super1300-*CsGH3.1*-Flag. (**b**) Phenotype of *CsGH3.1* overexpression lines. (**c**) Expression of *CsGH3.1*. (**d**) Seed germination. (**e**) Germination rate. (**f**) Root length. (**g**) Root phenotype. Values are means ± SD. ** *p* < 0.01 (Student’s *t* test).

To further investigate the functional role of *CsGH3.1* in the proliferation culture, a Super1300-*CsGH3.1*-GFP expression vector was constructed and used to transform *Arabidopsis* via the floral dip method. Three independent *Arabidopsis* overexpression lines (OE-1, OE-2, and OE-3) were obtained (Figure 4). Compared to wild-type *Arabidopsis*, the three overexpression lines exhibited delayed flowering and a dwarf phenotype (Figure 4b).

The seed germination and early seedling development of *CsGH3.1* overexpression lines were also significantly impaired. On 1/2 MS solid medium, wild-type seeds achieved a nearly 100% germination rate at 3 days post-sowing, while all three *CsGH3.1* overexpression lines showed significantly reduced germination rates of approximately 20% (Figure 4d,e). Furthermore, at 7 days post-sowing, the wild-type seedlings developed primary roots measuring 4.13 ± 0.07 cm in length. In contrast, the overexpression lines displayed significantly shortened primary roots, with lengths of 1.67 ± 0.15 cm, 1.60 ± 0.11 cm, and 1.50 ± 0.04 cm for the three independent transgenic lines, respectively (Figure 4f,g). These results collectively demonstrate that *CsGH3.1* overexpression significantly inhibits the early growth phases in *Arabidopsis*.

**Figure 5:**
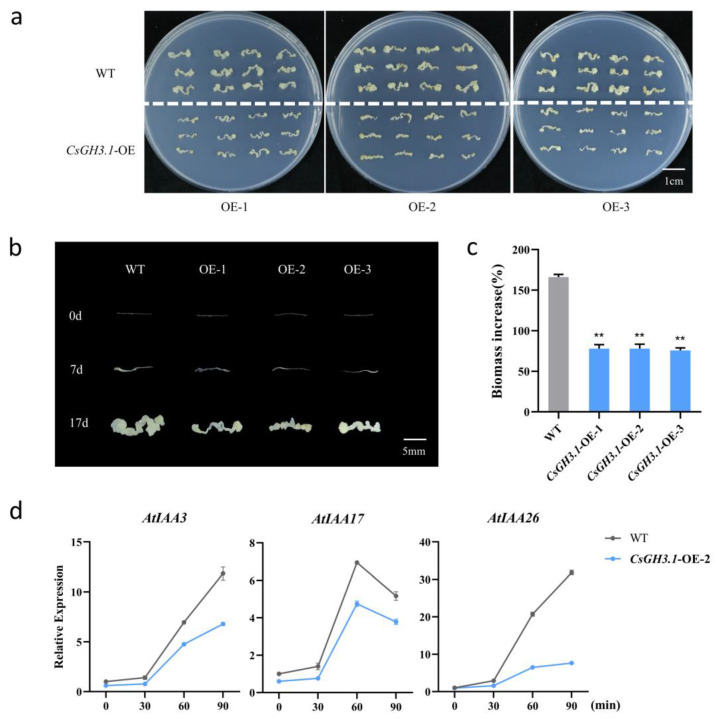
Proliferation characteristics of *CsGH3.1*-overexpression *Arabidopsis*. (**a**–**c**) Proliferation characteristics. (**d**) Expression of auxin-responsive genes. Values are means ± SD. ** *p* < 0.01 (Student’s *t* test).

The roots from 7-day-old seedlings of wild-type *Arabidopsis* and the three T3 generation *CsGH3.1* overexpression lines were used as explants to induce callus formation on the CIM medium (Figure 5). After 7 days, the callus was transferred to a 1/2 MS medium for 10 days of proliferation. All three *CsGH3.1* overexpression lines showed 80–90% reduced callus proliferation compared to the wild-type controls (Figure 5a–c). These results suggest that the overexpression of *CsGH3.1* in *Arabidopsis* negatively impacts plant growth and development, significantly reducing the proliferation efficiency of root explant-derived calluses.

Additionally, *CsGH3.1* overexpression exhibited distinct effects on the expression of *AtIAA3* in *Arabidopsis* (Figure 5d). While both the wild-type and *CsGH3.1*-OE-2 lines exhibited a rapid induction of *AtIAA3*, *AtIAA17*, and *AtIAA26* during culture initiation, the transcript levels of these genes were consistently attenuated in the *CsGH3.1*-OE-2 plants relative to their wild-type counterparts (Figure 5d). These findings suggest that *CsGH3.1* overexpression substantially impacts auxin signaling transduction in *Arabidopsis*, thereby reducing the plant’s sensitivity to auxin responses.

## 3. Discussion

As early auxin-responsive genes, the acyl-adenylate synthetase family *GH3* plays a crucial role in maintaining auxin homeostasis in plants [20,30,31,32,33]. Although *GH3* genes have been identified in many species, their functional characterization in orchids remains limited. Previous studies have identified 19 and 13 *GH3* family genes in *Arabidopsis* and rice, respectively [31]. In this study, seven *CsGH3* genes were identified from the transcriptome and genome of CSQ using bioinformatics methods. All *CsGH3* proteins contained conserved *GH3* domains and three conserved motifs. Based on their functional characteristics and sequence similarities, the *GH3* proteins in *Arabidopsis*, rice, maize, wheat, and potato are classified into three subfamilies [9,34,35,36]. In *Arabidopsis*, subfamily II members such as *AtGH3.1*, *AtGH3.2* (YDK), *AtGH3.3*, *AtGH3.4*, *AtGH3.5* (AtGH3a), and *AtGH3.6* (DFL1) catalyze the adenylation of IAA with amino acids [33,37]. Similarly, in rice, subfamily II members such as *OsGH3-1*, *OsGH3-2*, *OsGH3-8*, and *OsGH3-13* are involved in auxin signaling. Phylogenetic analysis revealed that the *CsGH3* genes in *CSQ* are clustered into subfamilies I and II (Figure 1), suggesting that subfamily II *CsGH3* genes may also participate in auxin signaling. The promoters of soybean *GH3* genes contain at least three auxin response elements (AuxREs) with a conserved TGTCTC sequence, which is essential for auxin responsiveness [38]. In this study, the promoters of four *CsGH3* genes in subfamily II contained core AuxREs, while those in subfamily I lacked these elements (Figure 2). These results suggest that subfamily II *CsGH3* genes are primarily involved in IAA signaling pathways.

*GH3* genes respond rapidly to auxin signals and catalyze the conjugation of free IAA with amino acids to form auxin conjugates, thereby reducing endogenous auxin levels and maintaining auxin homeostasis [5,16,39,40]. In *Arabidopsis thaliana*, subfamily II *AtGH3* genes, including *AtGH3.1*, *AtGH3.2*, *AtGH3.3*, and *AtGH3.4* [30,41], which cluster with *CsGH3.1* on the same evolutionary branch, are induced by auxin and are involved in synthesizing IAA-amido conjugates, thereby regulating auxin metabolic homeostasis. The expression of *AtGH3.9* is suppressed by low concentrations of exogenous IAA, and its overexpression results in shorter primary roots, indicating its primary role in regulating root growth and development [35]. In contrast, *AtGH3.17* is upregulated by auxin, and its enhanced expression is accompanied by increased levels of IAA-Glu and IAA-Asp. A loss of *AtGH3.17* function leads to an accumulation of free IAA, suggesting its critical role in maintaining auxin homeostasis by modulating conjugated auxin levels [2]. These results suggest that different GH3 genes may exhibit specialized functional roles in regulating auxin metabolism. In this study, the *CsGH3* subfamily II genes in *Cymbidium sinense* responded rapidly to auxin during rhizome proliferation, significantly increasing the conjugated auxin IAA-Asp (Figure 3c,d). Both *CsGH3.1* and *CsGH3.2* were rapidly induced by auxin, but *CsGH3.1* showed a faster and more pronounced increase in expression (Figure 3d). These findings suggest that *CsGH3.1* may play a more critical role in regulating auxin homeostasis during rhizome proliferation in *Cymbidium sinense*. It is proposed that *CsGH3.1* promotes the conjugation of IAA with amino acids, reducing free IAA levels and thereby regulating rhizome proliferation and development.

Maintaining active IAA levels is essential for normal plant growth and development. Previous studies have shown that overexpressing *GH3* genes results in auxin-deficient phenotypes and developmental abnormalities [42]. For example, in *Arabidopsis*, the auxin-responsive gene *AtGH3.6/dfl1-D* negatively regulates hypocotyl and lateral root elongation, resulting in a dwarf phenotype [35]. In rice, the overexpression of *OsGH3.8* and *OsGH3.13* causes slow growth and dwarfism, accompanied by increased levels of IAA-Asp and IAA-Glu conjugates [23,43]. In this study, three *CsGH3.1* overexpression *Arabidopsis* lines displayed dwarfism, delayed flowering, and reduced growth rates (Figure 4a), which is similar to the phenotypes observed in most *GH3* gene overexpression mutants. Similarly, the heterologous overexpression of *OsGH3.2* and *OsGH3.8* in *Arabidopsis* resulted in slow growth, dwarfism, and smaller leaves. During callus culture, callus proliferation was significantly reduced in all three *CsGH3.1* overexpression lines (Figure 4c–e). These results indicate that the overexpression of *CsGH3.1* causes growth abnormalities resembling auxin-deficient phenotypes, highlighting its critical role in regulating auxin levels and homeostasis. We propose that *CsGH3.1* modulates rhizome proliferation in *Cymbidium sinense* by conjugating auxin and reducing free IAA levels.

## 4. Materials and Methods

### 4.1. Identification and Bioinformatics Analysis of the GH3 Gene Family in CSQ

*GH3* gene family protein sequences from *Arabidopsis* and rice were retrieved from the NCBI database and were used for a BLAST 1.30 search against the transcriptomic and genomic databases of CSQ to preliminarily identify *GH3* genes. The presence of *GH3* protein domains in the candidate genes was confirmed by predicting conserved domains using the protein domain prediction tool (https://www.ncbi.nlm.nih.gov/Structure/cdd/wrpsb.cgi (accessed on 22 October 2023)). A phylogenetic tree was constructed using *MEGA7* to assess the homology of candidate genes with known *GH3* genes [44]. MEME was utilized to predict conserved motif elements in the identified *CsGH3s* (https://meme-suite.org/meme/index.html (accessed on 25 October 2023)). The 2 kb upstream region of the first exon of the *CsGH3s* was selected as the promoter region, and cis-elements were predicted using the PlantCARE website and visualized using TBtools v0.665 [45].

### 4.2. Rhizome Proliferation Culture of CSQ

The rhizomes of CSQ, which were originally induced from shoot apices and subsequently maintained through successive subcultures on a hormone-free MS medium under continuous darkness, served as the experimental material. The vigorous rhizomes of CSQ were excised, and the apical growth points were removed. The rhizomes were cut into approximately 1 cm segments and inoculated evenly onto the MS medium with or without 1.0 mg·L^−1^ NAA [26]. Each treatment was conducted with three biological replicates, with five bottles per replicate and ten rhizomes per bottle. After inoculation, the bottles were sealed with two layers of opaque black plastic bags and cultured in a 26 °C incubator for 30 days. The weight of the rhizomes was measured at the beginning and after 30 days of culture, and the morphology of the rhizomes was photographed for analysis.

### 4.3. Endogenous Hormone Measurement

Samples of the CSQ rhizomes grown under hormone-free and 1.0 mg·L^−1^ NAA conditions were collected at 0, 10, 20, and 30 days for endogenous hormone analysis. Ten rhizomes were randomly selected from each treatment, with three biological replicates. The samples were rapidly frozen in liquid nitrogen and stored at −80 °C. These samples were ground to a fine powder in liquid nitrogen using a mortar and pestle, then transferred to 10 mL centrifuge tubes with pre-chilled spatulas for fresh weight measurement. The quantification of endogenous auxins (IAA, IAA-Asp, and IAA-Glu) was conducted via ESI-HPLC-MS/MS using an Agilent 1290 UHPLC system coupled with electrospray ionization (ESI) and multiple reaction monitoring (MRM) (Table 2). Mass spectrometry data were processed using Analyst software 1.7 (Nanjing Ruiyuan Biotechnology Co., Ltd., Nanjing, China).

### 4.4. Expression Analysis of CsGH3 Genes

The CSQ rhizomes grown under hormone-free and 1.0 mg·L^−1^ NAA conditions were collected at 0, 10, 20, and 30 days. Six to seven rhizomes were randomly selected from each treatment, with three biological replicates. The samples were quickly frozen in liquid nitrogen and stored at −80 °C for a subsequent qPCR analysis of the gene expression patterns of their *CsGH3* genes.

Total RNA was extracted from the samples using the RNA Aprep Pure Plant Kit (Vazyme, Nanjing, China), following the manufacturer’s method. cDNAs were reverse transcribed using 5 HiScript III qRT SuperMix (Vazyme, Nanjing, China) and used for a subsequent quantitative analysis of candidate genes. The transcript levels of the genes were detected by qPCR using AceQ qPCR SYBR Green Master Mix (Vazyme) and the CFX96 Real-Time PCR Detection System (Bio-Rad, Hercules, CA, USA) in standard mode. The gene-specific primers are listed in Table 3, and the petunia *RPS3* gene was used as an internal control. The reaction conditions were 30 s at 95 °C and 40 cycles of 5 s at 95 °C and 30 s at 60 °C. Three biological replicates were performed in this study, and the expression data were quantified via the 2^−△△CT^ method [46].

### 4.5. Generation of Overexpressed-CsGH3.1 Arabidopsis Transgenic Materials

The CDS sequence of *CsGH3.1* was amplified and cloned into the Super1300-GFP vector to construct the overexpression vector. After *Arabidopsis* bolting, the opened flowers on the inflorescence were removed, leaving only the unopened flower buds. The inflorescence tip was immersed in the infection solution containing the plasmid for 3–5 s, after which the plants were kept in the dark overnight before being transferred to normal growth conditions. Approximately one month after transformation, dried and cracked siliques were collected, dried, and sown. Antibiotic selection was used to identify successful transformants. The genomic DNA of the selected transgenic lines was analyzed by PCR using hygromycin-specific primers (Hyg-F: 5′-GACATTGGGGAGTTTAGCGAGAGC-3′; Hyg-R: 5′-GGTGTCGTCCATCACAGTTTGCC-3′) to identify hygromycin-positive plants (Figure S1). The DNA of each sample was extracted using the CTAB method, and 2 μL was used as a template for PCR. PCR amplification was performed in a 50 µL reaction containing 2 µL primers, 25 µL 2 × Phanta Flash Master Mix (Dye Plus, Vazyme, Nanjing, China), and 19 µL ddH_2_O. The reaction conditions were 30 s at 98 °C, 30 cycles of 10 s at 98 °C, 5 s at 58 °C, 5 s at 72 °C, and finally, 60 s at 72 °C. RNA was extracted from the leaves of the T1 transgenic plants, and qPCR was performed to confirm the overexpression of *CsGH3.1* [47].

### 4.6. Phenotypic Analysis of CsGH3.1 Overexpression Arabidopsis Mutants

Three *CsGH3.1* overexpression *Arabidopsis* lines and a wild-type *Arabidopsis* were sown simultaneously, and their phenotypes were recorded after 50 days. T1 mutant seeds were surface-sterilized and cold-treated for 24 h before being germinated on 1/2 MS medium. After 7 days, the roots were cut into 1 cm segments and cultured on CIM medium (Gamborg’s B5 basal salts supplemented with 20.0 g·L^−1^ sucrose, 0.5 g·L^−1^ MES, 0.5 mg·L^−1^ 2,4-D, 0.05 mg·L^−1^ kinetin, and 5 g·L^−1^ agar, pH adjusted to 5.8) for 7 days to induce callus formation. The callus was then transferred to a hormone-free B5 medium for proliferation, and the biomass increment was measured at 0 and 10 days [48].

### 4.7. Statistical Analysis

Biomass increment of rhizomes, endogenous hormone levels, and relative gene expression levels were analyzed from three biological replicates. Data were processed using GraphPad to calculate least significant difference at *p* = 0.05, and results were expressed as mean ± standard error of mean (SEM) (n = 3). Statistical significance was determined at *p* = 0.05 [47].

## 5. Conclusions

This study represents the first systematic identification of the *GH3* gene family in Cymbidium sinense and explores its potential roles in auxin and jasmonic acid metabolism. Evolutionary analysis revealed that *CsGH3* genes are divided into two subfamilies. Gene structure and cis-element analyses highlighted the significant role of *CsGH3* genes in hormone responses. *CsGH3.1* was significantly upregulated in response to auxin during rhizome proliferation in CSQ, suggesting its key role in regulating auxin homeostasis. The overexpression of *CsGH3.1* in Arabidopsis significantly inhibited callus proliferation, confirming its involvement in cell proliferation and development, as well as in auxin homeostasis. These findings enhance our understanding of the functional roles of the *GH3* gene family and provide a theoretical foundation for molecular breeding and hormone regulation research for *Cymbidium sinense*. Future studies should focus on the functional validation of *CsGH3.1* and its regulatory networks in rhizome development, offering insights for orchid breeding and cultivation.

## Figures and Tables

**Table 1:** List of identified *CsGH3* genes in *CSQ*.

Gene Name	Gene Transcriptome ID	Gene Locus ID	Chr	Location	CDS (bp)	Protein (aa)	MW (Da)	pI	Formula
CsGH3.1	Unigene0063066	CYSIN007746	15	38,528,251–38,530,808	1806	602	67,940.94	5.79	C3037H4761N815O893S30
CsGH3.2	Unigene0045160	CYSIN015084	9	47,807,899–47,810,722	1791	597	66,682.16	5.61	C2981H4642N800O881S28
CsGH3.3	Unigene0023145	CYSIN028796	9	62,449,145–62,453,024	1833	611	68,633.54	5.59	C3052H4805N829O910S30
CsGH3.4	Unigene0031003	CYSIN005890	2	258,273,160–258,276,267	1815	605	67,626.52	5.68	C3010H4723N807O895S34
CsGH3.5	Unigene0075990	CYSIN028599	14	45,363,266–45,384,562	1821	607	68,287.87	6.27	C3064H4774N826O902S21
CsGH3.6	Unigene0020803	CYSIN006822	8	120,446,007–120,448,018	1758	586	65,523.81	6.2	C2943H4587N779O871S22
CsGH3.7	Unigene0031127	CYSIN027452	13	72,632,018–72,639,833	1761	587	65,895.12	6.22	C2957H4604N782O880S22

**Table 2:** Selected reaction monitoring conditions for protonated and deprotonated plant hormones ([M + H]^+^ or [M − H]^−^).

Hormone Name	Polarity	Parent Ion (*m*/*z*)	Daughter Ion (*m*/*z*)	Breakdown Voltage (V)	Collisional Energy (V)
IAA-ASP	+	291.1	134/130.1 *	110	8/35
IAA-GLU	−	303.0	146.0 */127.8/173.9	−40	−23/−25/−20
IAA	+	176.2	129.8 */102.9	65	12/42

Note: Labeled * is quantitative ion.

**Table 3:** Primers for qRT-PCR.

Primer Name	Primer Sequence (5′-3′)
RPS3-F	TGAAGCCAATGCAGAACAAACG
RPS3-R	ATGGATTTCACCACCGACAAGC
qP-BMGH3.1-F	TCTCCTTATGCCTGTTATG
qP-BMGH3.1-R	GAAATCGGAAACCAGAACT
qP-BMGH3.2-F	AGGCGACAATCACAACCAA
qP-BMGH3.2-R	CCGATACCGATACAAACCG
qP-BMGH3.3-F	GAACATCAGCGGGAGAGAG
qP-BMGH3.3-R	ACGAAGAGGAAGTAAAGGG
qP-BMGH3.4-F	TAGAGGCTGTGCTTACTGG
qP-BMGH3.4-R	GATGATGCATACATCGTGC
qP-BMGH3.5-F	CGTTGTATTGCCACCTTCT
qP-BMGH3.5-R	TTCTTCCCAAACATTCTCG
qP-BMGH3.6-F	ATCGAGCAGAGAGAGACAG
qP-BMGH3.6-R	AAACCTCCAAAAGTAGTGA
qP-BMGH3.7-F	TAGAGGCTGTGCTTACTGG
qP-BMGH3.7-R	GATGATGCATACATCGTGC

## Data Availability

The genomic data mentioned in our article are available on GenBank under the accession number SAN20059972. The transcriptome data can be accessed through the NCBI “Short Read Archive” (SRA) under the accession number SRP073228.

## Supplementary Materials

The following supporting information can be downloaded at https://www.mdpi.com/article/10.3390/plants14091287/s1, Figure S1. PCR detection of the *hygromycin* gene in *CsGH3.1* overexpression *Arabidopsis* lines. M: DL2000 DNA marker; Lane 1: amplification product from wild-type *Arabidopsis* plants; Lanes 2–4: amplification products from independent *CsGH3.1*-OE transgenic lines (OE-1, OE-2, and OE-3).

## Abbreviations

The following abbreviations are used in this manuscript:

CSQ	*Cymbidium sinense* ‘Qijianbaimo’
IAA	indoleacetic acid
NAA	naphthaleneacetic acid
IBA	indolebutyric acid

## References

[1] Woodward A.W., Bartel B. (2005). Auxin: Regulation, action, and interaction. Ann. Bot..

[2] Zheng Z., Guo Y., Novák O., Chen W., Ljung K., Noel J.P., Chory J. (2016). Local auxin metabolism regulates environment-induced hypocotyl elongation. Nat. Plants.

[3] Mateo-Bonmatí E., Casanova-Sáez R., Šimura J., Ljung K. (2021). Broadening the roles of UDP-glycosyltransferases in auxin homeostasis and plant development. New Phytol..

[4] Westfall C.S., Zubieta C., Herrmann J., Kapp U., Nanao M.H., Jez J.M. (2012). Structural basis for prereceptor modulation of plant hormones by GH3 proteins. Science.

[5] Luo P., Li T.-T., Shi W.-M., Ma Q., Di D.-W. (2023). The roles of GRETCHEN HAGEN3 (GH3)-dependent auxin conjugation in the regulation of plant development and stress adaptation. Plants.

[6] Staswick P.E., Serban B., Rowe M., Tiryaki I., Maldonado M.T., Maldonado M.C., Suza W. (2005). Characterization of an Arabidopsis enzyme family that conjugates amino acids to indole-3-acetic acid. Plant Cell.

[7] Hagen G., Guilfoyle T. (2002). Auxin-responsive gene expression: Genes, promoters and regulatory factors. Plant Mol. Biol..

[8] Fu J., Yu H., Li X., Xiao J., Wang S. (2011). Rice GH3 gene family: Regulators of growth and development. Plant Signal. Behav..

[9] Jain M., Kaur N., Tyagi A.K., Khurana J.P. (2006). The auxin-responsive GH3 gene family in rice (*Oryza sativa*). Funct. Integr. Genom..

[10] Kong W., Zhang Y., Deng X., Li S., Zhang C., Li Y. (2019). Comparative genomic and transcriptomic analysis suggests the evolutionary dynamic of GH3 genes in Gramineae crops. Front. Plant Sci..

[11] Pinto R.T., Freitas N.C., Máximo W.P.F., Cardoso T.B., de Oliveira Prudente D., Paiva L.V. (2019). Genome-wide analysis, transcription factor network approach and gene expression profile of GH3 genes over early somatic embryogenesis in *Coffea* spp.. BMC Genom..

[12] Monsalve L., Ayala-Raso A., Bernales M., Valdenegro M., Defilippi B., González-Agüero M., Cherian S., Fuentes L. (2018). Dataset on quality and physiological changes of raspberry fruit during their development and under auxin in-vitro assay. Data Brief..

[13] Terol J., Domingo C., Talón M. (2006). The GH3 family in plants: Genome wide analysis in rice and evolutionary history based on EST analysis. Gene.

[14] Kumar R., Agarwal P., Tyagi A.K., Sharma A.K. (2012). Genome-wide investigation and expression analysis suggest diverse roles of auxin-responsive GH3 genes during development and response to different stimuli in tomato (*Solanum lycopersicum*). Mol. Genet. Genom..

[15] Nakazawa M., Yabe N., Ichikawa T., Yamamoto Y.Y., Yoshizumi T., Hasunuma K., Matsui M. (2001). DFL1, an auxin-responsive GH3 gene homologue, negatively regulates shoot cell elongation and lateral root formation, and positively regulates the light response of hypocotyl length. Plant J..

[16] Park J.-E., Park J.-Y., Kim Y.-S., Staswick P.E., Jeon J., Yun J., Kim S.-Y., Kim J., Lee Y.-H., Park C.-M. (2007). GH3-mediated auxin homeostasis links growth regulation with stress adaptation response in Arabidopsis. J. Biol. Chem..

[17] Park J.-E., Seo P.J., Lee A.-K., Jung J.-H., Kim Y.-S., Park C.-M. (2007). An Arabidopsis GH3 gene, encoding an auxin-conjugating enzyme, mediates phytochrome B-regulated light signals in hypocotyl growth. Plant Cell Physiol..

[18] Takase T., Nakazawa M., Ishikawa A., Kawashima M., Ichikawa T., Takahashi N., Shimada H., Manabe K., Matsui M. (2004). ydk1-D, an auxin-responsive GH3 mutant that is involved in hypocotyl and root elongation. Plant J..

[19] Domingo C., Andrés F., Tharreau D., Iglesias D.J., Talón M. (2009). Constitutive expression of OsGH3. 1 reduces auxin content and enhances defense response and resistance to a fungal pathogen in rice. Mol. Plant-Microbe Interact..

[20] Du H., Wu N., Fu J., Wang S., Li X., Xiao J., Xiong L. (2012). A GH3 family member, OsGH3-2, modulates auxin and abscisic acid levels and differentially affects drought and cold tolerance in rice. J. Exp. Bot..

[21] Yuan Z., Fan K., Wang Y., Tian L., Zhang C., Sun W., He H., Yu S. (2021). OsGRETCHENHAGEN3-2 modulates rice seed storability via accumulation of abscisic acid and protective substances. Plant Physiol..

[22] Dai Z., Wang J., Yang X., Lu H., Miao X., Shi Z. (2018). Modulation of plant architecture by the miR156f–OsSPL7–OsGH3. 8 pathway in rice. J. Exp. Bot..

[23] Zhang S.-W., Li C.-H., Cao J., Zhang Y.-C., Zhang S.-Q., Xia Y.-F., Sun D.-Y., Sun Y. (2009). Altered architecture and enhanced drought tolerance in rice via the down-regulation of indole-3-acetic acid by TLD1/OsGH3. 13 activation. Plant Physiol..

[24] Yadav S.R., Khanday I., Majhi B.B., Veluthambi K., Vijayraghavan U. (2011). Auxin-responsive OsMGH3, a common downstream target of *OsMADS1* and *OsMADS6*, controls rice floret fertility. Plant Cell Physiol..

[25] Li X., Yang D.-L., Sun L., Li Q., Mao B., He Z. (2016). The systemic acquired resistance regulator OsNPR1 attenuates growth by repressing auxin signaling through promoting IAA-amido synthase expression. Plant Physiol..

[26] Liu Y., Zhang H.-L., Guo H.-R., Xie L., Zeng R.-Z., Zhang X.-Q., Zhang Z.-S. (2017). Transcriptomic and hormonal analyses reveal that YUC-mediated auxin biogenesis is involved in shoot regeneration from rhizome in *Cymbidium*. Front. Plant Sci..

[27] Xu C., Cao H., Zhang Q., Wang H., Xin W., Xu E., Zhang S., Yu R., Yu D., Hu Y. (2018). Control of auxin-induced callus formation by bZIP59–LBD complex in Arabidopsis regeneration. Nat. Plants.

[28] Yang F.-X., Gao J., Wei Y.-L., Ren R., Zhang G.-Q., Lu C.-Q., Jin J.-P., Ai Y., Wang Y.-Q., Chen L.-J. (2021). The genome of Cymbidium sinense revealed the evolution of orchid traits. Plant Biotechnol. J..

[29] Hu W., Fagundez S., Katin-Grazzini L., Li Y., Li W., Chen Y., Wang X., Deng Z., Xie S., McAvoy R.J. (2017). Endogenous auxin and its manipulation influence in vitro shoot organogenesis of citrus epicotyl explants. Hortic. Res..

[30] Wojtaczka P., Ciarkowska A., Starzynska E., Ostrowski M. (2022). The GH3 amidosynthetases family and their role in metabolic crosstalk modulation of plant signaling compounds. Phytochemistry.

[31] Bao D., Chang S., Li X., Qi Y. (2024). Advances in the study of auxin early response genes: Aux/IAA, GH3, and SAUR. Crop J..

[32] Jiang W., Yin J., Zhang H., He Y., Shuai S., Chen S., Cao S., Li W., Ma D., Chen H. (2020). Genome-wide identification, characterization analysis and expression profiling of auxin-responsive GH3 family genes in wheat (*Triticum aestivum* L.). Mol. Biol. Rep..

[33] Takase T., Nakazawa M., Ishikawa A., Manabe K., Matsui M. (2003). DFL2, a new member of the Arabidopsis GH3 gene family, is involved in red light-specific hypocotyl elongation. Plant Cell Physiol..

[34] Feng S., Yue R., Tao S., Yang Y., Zhang L., Xu M., Wang H., Shen C. (2015). Genome-wide identification, expression analysis of auxin-responsive GH3 family genes in maize (*Zea mays* L.) under abiotic stresses. J. Integr. Plant Biol..

[35] Khan S., Stone J.M. (2007). *Arabidopsis thaliana* GH3. 9 influences primary root growth. Planta.

[36] Yan W., Jian Y., Duan S., Guo X., Hu J., Yang X., Li G. (2023). Dissection of the plant hormone signal transduction network in late blight-resistant potato genotype SD20 and prediction of key resistance genes. Phytopathology®.

[37] Nobuta K., Okrent R.A., Stoutemyer M., Rodibaugh N., Kempema L., Wildermuth M.C., Innes R.W. (2007). The GH3 acyl adenylase family member PBS3 regulates salicylic acid-dependent defense responses in Arabidopsis. Plant Physiol..

[38] Liu Z.-B., Ulmasov T., Shi X., Hagen G., Guilfoyle T.J. (1994). Soybean GH3 promoter contains multiple auxin-inducible elements. Plant Cell.

[39] Ai G., Huang R., Zhang D., Li M., Li G., Li W., Ahiakpa J.K., Wang Y., Hong Z., Zhang J. (2023). SlGH3. 15, a member of the GH3 gene family, regulates lateral root development and gravitropism response by modulating auxin homeostasis in tomato. Plant Sci..

[40] Zhao D., Wang Y., Feng C., Wei Y., Peng X., Guo X., Guo X., Zhai Z., Li J., Shen X. (2020). Overexpression of MsGH3. 5 inhibits shoot and root development through the auxin and cytokinin pathways in apple plants. Plant J..

[41] Hayashi K.-i., Arai K., Aoi Y., Tanaka Y., Hira H., Guo R., Hu Y., Ge C., Zhao Y., Kasahara H. (2021). The main oxidative inactivation pathway of the plant hormone auxin. Nat. Commun..

[42] Yu D., Qanmber G., Lu L., Wang L., Li J., Yang Z., Liu Z., Li Y., Chen Q., Mendu V. (2018). Genome-wide analysis of cotton GH3 subfamily II reveals functional divergence in fiber development, hormone response and plant architecture. BMC Plant Biol..

[43] Ding X., Cao Y., Huang L., Zhao J., Xu C., Li X., Wang S. (2008). Activation of the indole-3-acetic acid–amido synthetase GH3-8 suppresses expansin expression and promotes salicylate-and jasmonate-independent basal immunity in rice. Plant Cell.

[44] Kumar S., Stecher G., Tamura K. (2016). MEGA7: Molecular evolutionary genetics analysis version 7.0 for bigger datasets. Mol. Biol. Evol..

[45] Chen C., Chen H., Zhang Y., Thomas H.R., Frank M.H., He Y., Xia R. (2020). TBtools: An integrative toolkit developed for interactive analyses of big biological data. Mol. Plant.

[46] Bin J., Tan Q., Wen S., Huang L., Wang H., Imtiaz M., Zhang Z., Guo H., Xie L., Zeng R. (2024). Comprehensive Analyses of Four *PhNF-YC* Genes from *Petunia hybrida* and Impacts on Flowering Time. Plants.

[47] Dai X., Wang J., Wang L., Liu Z., Li Q., Cai Y., Li S., Xiang F. (2022). HY5 inhibits in vitro shoot stem cell niches initiation via directly repressing pluripotency and cytokinin pathways. Plant J..

[48] Mallona I., Lischewski S., Weiss J., Hause B., Egea-Cortines M. (2010). Validation of Reference Genes for Quantitative Real-Time PCR during Leaf and Flower Development in *Petunia hybrida*. BMC Plant Biol..

