# Supplementary material for: Genome-Wide Analysis of GRETCHEN HAGEN3 Genes and Characterization of IAA-Amido Synthetase Gene CsGH3.1 in Rhizome Proliferation in Cymbidium sinense ‘Qijianbaimo’
Source: Plants (Basel). 2025 Apr 24;14(9):1287. doi: 10.3390/plants14091287 (PMC12073139; doi:10.3390/plants14091287)
Supplement: Supplementary file 1 [file plants-14-01287-s001.zip › plants-3580524-supplementary.pdf]

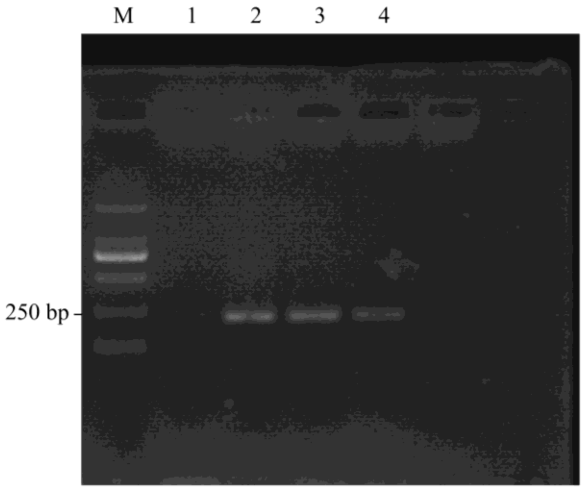

Figure S1. PCR detection of the *hygromycin* gene in *CsGH3.1* overexpression *Arabidopsis* lines.

M: DL2000 DNA marker; Lane 1: amplification product from wild-type *Arabidopsis* plants; Lanes 2-4: amplification products from independent *CsGH3.1*-OE transgenic lines (OE-1, OE-2, and OE-3).
